# Routine Prescribing, Unexpected Consequence: Takotsubo Cardiomyopathy Following Therapeutic Venlafaxine Dose Escalation

**DOI:** 10.1192/bjo.2026.11764

**Published:** 2026-06-30

**Authors:** Sheikh Aslam, Nigel Ashurst, Assmaa Hussein

**Affiliations:** Kent and Medway Mental Health NHS Trust, Kent, United Kingdom

## Abstract

**Aims:**

Takotsubo cardiomyopathy is an acute condition characterised by transient left ventricular systolic dysfunction, often triggered by severe physical and emotional stress, presenting with an acute coronary syndrome-like presentation but with normal coronaries. It has been associated in multiple reports with serotonin-noradrenaline reuptake inhibitors, most commonly venlafaxine, specifically in overdose and postmenopausal women, with fewer cases following routine dose escalation or younger adults. This case illustrates the combined influence of medication exposure, underlying autonomic vulnerabilities, and psychosocial stressors in the development of Takotsubo cardiomyopathy.

**Methods:**

A woman in her early-30s with a background of mixed anxiety and depression, functional neurological disorder (FND), and postural orthostatic tachycardia syndrome (POTS) was admitted following an overdose of mirtazapine. Mirtazapine was discontinued following bradycardia and hypotension; venlafaxine modified release 75 mg was commenced during admission and increased to 150 mg during routine Home Treatment Team follow-up. Clinical review identified high consumption of energy drinks. Seven weeks following dose titration, she developed acute severe chest pain and three witnessed non-epileptic seizures requiring urgent medical assessment. Investigations revealed 3865 ng/L troponin without acute ST-T wave changes on ECG; however, echocardiography showed ejection fraction of 40% ± 5% with regional wall motion abnormalities. Cardiology diagnosed Takotsubo cardiomyopathy, as confirmed by cardiac MRI. She was managed with beta-blockers and angiotensin-converting enzyme inhibitors and subsequently discharged home with follow-up in 6 months for repeat cardiac MRI.

**Results:**

The patient was diagnosed with Takotsubo cardiomyopathy and demonstrated clinical improvement following venlafaxine cessation and heart failure management. This presentation is atypical, both in terms of the patient’s age and occurrence following routine dose escalation within therapeutic limits. Existing evidence associates Takotsubo cardiomyopathy primarily with venlafaxine overdose and in postmenopausal women. The convergence of venlafaxine at a dose of 150mg, at which noradrenaline reuptake inhibition becomes clinically relevant, together with high caffeine intake from energy drinks, and preexisting autonomic dysfunction POTS and FND likely contributed to a state of synergistic catecholamine excess, consistent with myocardial stunning. This case supports a multifactorial understanding of Takotsubo cardiomyopathy, highlighting the interaction between physiological vulnerability and external stressors.

**Conclusion:**

This case highlights the importance of considering underlying physical health vulnerabilities, including autonomic dysregulation, during routine psychopharmacological prescribing and dose titration, with heightened vigilance for cardiac symptoms when escalating venlafaxine in at-risk patients, even in the absence of formal contraindications.

